# Indikationsspektrum zur Lungentransplantation – Weiterentwicklungen seit den letzten ISHLT-Empfehlungen

**DOI:** 10.1055/a-2563-3691

**Published:** 2025-05-15

**Authors:** Zsofia Kovacs, Alberto Benazzo, Peter Jaksch

**Affiliations:** 1 27271 Thoraxchirurgie, Medizinische Universität Wien, Wien, Österreich

**Keywords:** Transplantation, Immunsuppression, interdisziplinär, transplantation, immune suppression, interdisciplinary

## Abstract

Die Lungentransplantation hat sich seit ihren ersten erfolgreichen Verfahren in den 1960er-Jahren kontinuierlich weiterentwickelt. Die aktuellen Empfehlungen der International Society for Heart and Lung Transplantation (ISHLT) betonen eine zunehmend individualisierte Patientenbewertung, die neben der zugrunde liegenden Lungenerkrankung auch Faktoren wie Begleiterkrankungen, Gebrechlichkeit, Alter und soziale Aspekte berücksichtigt. Die erweiterten Indikationen für Lungentransplantationen spiegeln sich in der verfeinerten Risikobewertung wider, die insbesondere Patienten mit fortgeschrittener chronisch obstruktiver Lungenerkrankung (COPD), idiopathischer pulmonaler Fibrose (IPF) und pulmonaler arterieller Hypertonie (PAH) einbezieht. Zudem wurden die Kriterien für Patienten mit einer Krebserkrankung in der Vorgeschichte sowie solchen mit Infektionen wie HIV oder multiresistenten Erregern flexibilisiert, was zu einer inklusiveren Transplantationspolitik führt. Ein wesentlicher Schwerpunkt liegt auf der frühzeitigen Transplantationsberatung, um den Patienten vor der Entwicklung von akuten Exazerbationen die Möglichkeit einer Transplantation zu eröffnen. Diese aktualisierten Richtlinien zielen darauf ab, sowohl die Überlebensraten als auch die Lebensqualität der Transplantationspatienten durch eine differenzierte und risikoangepasste Entscheidungsfindung zu maximieren.

## Abstract (en)

Lung transplantation has evolved continuously since its first successful procedures in the 1960 s. The current guidelines from the International Society for Heart and Lung Transplantation (ISHLT) emphasise increasingly individualised patient assessment, which, in addition to the underlying lung disease, considers factors such as comorbidities, frailty, age, and social aspects. The expanded indications for lung transplantation are reflected in the refined risk assessment, which particularly includes patients with advanced chronic obstructive pulmonary disease (COPD), idiopathic pulmonary fibrosis (IPF), and pulmonary arterial hypertension (PAH). Furthermore, the criteria for patients with a history of cancer and those with infections such as HIV or multidrug-resistant organisms have been made more flexible, leading to a more inclusive transplantation policy. A key focus is on early transplant counselling, allowing patients the opportunity for transplantation before they develop acute exacerbations. These updated guidelines aim to maximise both the survival rates and the quality of life of transplant patients, through differentiated and risk-adjusted decision-making.

## Einleitung

Die Lungentransplantation stellt einen bedeutenden Fortschritt in der modernen Medizin dar und hat sich seit den ersten erfolgreichen Eingriffen in den späten 1960er-Jahren kontinuierlich weiterentwickelt. Der erste Lungentransplantationseingriff wurde 1963 von Dr. James D. Hardy an der University of Mississippi durchgeführt. Der Patient überlebte jedoch nur wenige Tage. Doch trotz dieser frühen Rückschläge markierte dieser Eingriff den Beginn einer Ära, die von bahnbrechenden Fortschritten in der Chirurgie, der Medikation und der Transplantationswissenschaft geprägt war. In den 1980er-Jahren kam die Einführung von Immunsuppressiva, insbesondere des Medikaments Ciclosporin, als ein wesentlicher Durchbruch. Die verbesserte Kontrolle der Abstoßungsreaktionen führte zu einer signifikanten Erhöhung der Überlebensraten nach Lungentransplantationen, was die Anwendung der Transplantation bei Patienten mit schweren, nicht mehr behandelbaren Lungenerkrankungen ermöglichte.

Zwar gehören nach wie vor die chronisch obstruktive Lungenerkrankung (COPD) und die interstitielle Lungenerkrankung (ILD) zu den häufigsten Gründen für eine Transplantation, jedoch wurden die Indikationen zunehmend angepasst. Zudem konnte der Donorpool durch verbesserte Organerhaltungstechniken und erweiterte Transplantationsrichtlinien erheblich vergrößert werden 1 . Diese Veränderungen spiegeln sich auch in den fortlaufenden Empfehlungen der International Society for Heart and Lung Transplantation (ISHLT) wider 2 3 4 .

Die ISHLT-Richtlinien, zuletzt im Jahr 2021 überarbeitet 3 , definieren erweiterte medizinische und soziale Faktoren der Patienten, die für eine Lungentransplantation in Betracht gezogen werden. Ein zentrales Anliegen der aktualisierten Richtlinien ist die Individualisierung der Patientenbewertung, um nicht nur die Überlebensraten, sondern auch die Lebensqualität und die funktionellen Ergebnisse nach der Transplantation zu maximieren. Die zunehmende Anerkennung von Faktoren wie Alter, Begleiterkrankungen, allgemeinem Gesundheitszustand und dem sozialen Hintergrund des Patienten hat die Entscheidungskriterien für die Transplantation erheblich verändert.

Die Entscheidung über eine Lungentransplantation ist heutzutage nicht mehr ausschließlich auf das Vorliegen der zugrunde liegenden Lungenerkrankung beschränkt, sondern berücksichtigt vielmehr eine Vielzahl von Faktoren, die den klinischen Verlauf und den langfristigen Erfolg der Transplantation beeinflussen können. Die wichtigsten Entwicklungen in den letzten Jahren betreffen daher vor allem die Einschätzung von Risikofaktoren, wie etwa Begleiterkrankungen, die Gebrechlichkeit des Patienten (Frailty) 5 , den sozialen Hintergrund und die Lebensqualität des Patienten.

In Bezug auf Infektionskrankheiten, wie etwa multiresistente Erreger oder chronische Virusinfektionen (Humanes Immundefizienz-Virus [HIV], Hepatitis), berücksichtigt die neue Richtlinie die Fortschritte in der medizinischen Behandlung, die mittlerweile auch bei diesen Patienten erfolgreiche Transplantationsergebnisse ermöglichen 6 . Dies führt zu einer inklusiveren Haltung gegenüber solchen Patientengruppen 7 8 .

Ein weiterer Fortschritt in den neuen Richtlinien betrifft die Handhabung von Krebserkrankungen in der Vorgeschichte von Transplantationskandidaten. Früher wurden Patienten mit einer Tumorerkrankung oft pauschal von der Transplantation ausgeschlossen. Heute wird jedoch ein differenzierterer Ansatz verfolgt, der je nach Art des Tumors und dem Rezidivrisiko eine individuellere Entscheidungsfindung ermöglicht 9 .

## Altersgrenze

Ein wichtiger Punkt der aktualisierten Richtlinien bezieht sich auf die Altersgrenze für die Lungentransplantation. Während frühere Richtlinien bei Patienten über 70 Jahren eher von einer Lungentransplantation abrieten, zeigen die neuesten Daten, dass sorgfältig ausgewählte ältere Patienten auch nach einer Lungentransplantation vergleichbare Ergebnisse wie jüngere Patienten erzielen können. Es wird erwartet, dass die Ergebnisse bei älteren Patienten durch eine sorgfältige Risikobewertung und Optimierung der Begleiterkrankungen weiter verbessert werden können. Auch wenn Empfänger über 70 Jahre ein geringeres Langzeitüberleben aufweisen, sollte das biologische Alter zusammen mit den anderen Risikofaktoren bewertet werden 10 11 .

## Chronisch obstruktive Lungenerkrankung

Insbesondere bei Patienten mit schwerer chronisch obstruktiver Lungenerkrankung (COPD) wird ein zunehmend differenzierterer Ansatz verfolgt. Die ISHLT-Richtlinien empfehlen, dass Patienten mit fortgeschrittener COPD, die jedoch noch nicht unmittelbar gefährdet sind, frühzeitig eine spezialisierte Transplantationsberatung erhalten. Dies betrifft insbesondere Patienten, bei denen zusätzlich pulmonale Hypertonie, Hyperkapnie, schwere Exazerbationen oder eine schnelle Verschlechterung der Lungenerkrankung festgestellt werden. Bei bestimmten Patienten mit COPD kann eine Lungenvolumenreduktion (LVR) vorübergehend zu einer Verbesserung des Krankheitsverlaufs führen, ohne dass dies langfristig negative Auswirkungen auf das Überleben nach einer späteren Transplantation hat. Es ist jedoch Vorsicht bei der Auswahl dieser Patienten geboten, um mögliche postoperative Komplikationen wie Infektionen zu minimieren 12 .

## Verdächtige pulmonale Rundherde

Patienten mit verdächtigen pulmonalen Rundherden sollten nicht grundsätzlich von einer Lungentransplantation (LuTx) ausgeschlossen werden. In einer retrospektiven Analyse wurden bei 6,9% der LuTx-Patienten solche Rundherde festgestellt, von denen sich lediglich 17,3% als maligne erwiesen. Selbst bei Nachweis eines Frühstadiums eines Lungenkarzinoms in der explantierten Lunge zeigte sich ein akzeptables Langzeitüberleben. Die 5-Jahres-Überlebensrate betrug 73,1% für Patienten ohne verdächtige Rundherde und 59,2% bei Patienten mit bestätigtem Malignom 13 . Eine engmaschige bildmorphologische Kontrolle, z. B. im Rahmen der 3-monatlichen LAS-Updates (LAS: Lung Allocation Score), ist jedoch empfehlenswert.

## Idiopathische pulmonale Fibrose

Die idiopathische pulmonale Fibrose (IPF) stellt eine weitere häufige Indikation für Lungentransplantationen dar. Antifibrotische Medikamente wie Nintedanib und Pirfenidon haben in den letzten Jahren den Krankheitsverlauf verlangsamt und auch die Zahl der akuten Exazerbationen reduziert. Dennoch bleibt die Transplantation für Patienten mit fortgeschrittener IPF oft die einzige Option, da diese Medikamente nicht direkt die Mortalität beeinflussen. In diesem Zusammenhang empfiehlt die ISHLT weiterhin eine frühe Überweisung von IPF-Patienten zur Transplantationsbewertung, um ihnen die Möglichkeit zu geben, von einer Lungentransplantation zu profitieren, bevor eine akute Exazerbation auftritt. Patienten mit Bindegewebserkrankungen (CTD-ILD: Connective Tissue Diseases – z. B. systemische Sklerose [Sklerodermie], rheumatoide Arthritis, Dermatomyositis/Polymyositis und Mischkollagenose) wird eine Lungentransplantation grundsätzlich nicht verweigert, jedoch sollte eine gründliche und sorgfältige Evaluierung durchgeführt werden 2 .

## Acute Respiratory Distress Syndrome

Ein weiterer zentraler Aspekt der ISHLT-Richtlinien ist der Einsatz von Lungentransplantationen bei Patienten mit akutem Lungenversagen (ARDS), insbesondere infolge schwerer Virusinfektionen wie COVID-19. Seit 2020 wurden bilaterale Lungentransplantationen für COVID-19-assoziiertes ARDS dokumentiert 14 . Wann der richtige Zeitpunkt für eine LuTX bei ARDS ist, wird immer noch kontrovers diskutiert. Es wird empfohlen, mindestens 4–6 Wochen nach Beginn des Atemversagens abzuwarten, bevor eine Transplantation erwogen wird. In Deutschland stellt das „Bridging to Transplantation“ für Patienten, die zuvor nicht umfassend evaluiert wurden, eine Abweichung von den bestehenden Richtlinien dar. Solche Entscheidungen müssen gemäß den Vorgaben der Bundesärztekammer (BÄK) gemeldet werden.

In einer Analyse von Daten aus 3 großen europäischen Transplantationszentren zeigte sich, dass Virusinfektionen die Hauptursache für ARDS waren. Alle Patienten benötigten mechanische Beatmung und ECMO-Unterstützung. Die postoperative Beatmungsdauer betrug im Median 33 Tage, und 30 Tage nach der Transplantation lag die Mortalität bei 7,7%. Die 1- und 5-Jahres-Überlebensraten betrugen 71,6% bzw. 54,2%. Auch wenn die Überlebensraten schlechter sind als in der normalen Lungentransplantationskohorte, sollte die LuTX als lebensrettende Option für sorgfältig ausgewählte ARDS-Patienten angesichts fehlender alternativer Behandlungsmöglichkeiten nicht vorenthalten werden 15 .

Faktoren für ein positives Ergebnis nach Transplantation:

- jüngeres Alter
- keine Vorerkrankungen
- keine extra-pulmonalen Organdysfunktionen
- Verwendung von ECLS (Extracorporeal Life Support) als Brücke zur Transplantation
- pulmonale Ursache des ARDS

Die Transplantation und perioperative Betreuung sind jedoch komplex und sollten nur in erfahrenen Zentren durchgeführt werden. Im Optimalfall erfolgt die Behandlung unter „Awake ECMO“, wobei der Patient während der extrakorporalen Membranoxygenierung wach bleibt, was eine schnellere Mobilisierung und eine Prähabilitation ermöglicht.

## Mukoviszidose

Die Mukoviszidose (CF) stellte ebenfalls eine wichtige Indikation für Lungentransplantationen dar. Zwar hat die Einführung von hochwirksamen Medikamenten wie Elexacaftor/Tezacaftor/Ivacaftor (ETI) in den letzten Jahren die Zahl der Lungentransplantationen bei CF-Patienten signifikant verringert, jedoch bleibt die Lungentransplantation weiterhin relevant für Patienten, deren Krankheit trotz der Therapie bei selteneren Mutationen oder Therapieabbruch/Unverträglichkeit fortschreitet 16 .

## Pulmonale arterielle Hypertonie

Ein weiteres bedeutendes Thema ist die pulmonale arterielle Hypertonie (PAH), bei der die bilaterale Lungentransplantation für therapieresistente Erkrankungen als bevorzugte Methode für die Behandlung gilt. Die bilaterale Lungentransplantation erzielt ähnliche Ergebnisse wie eine kombinierte Herz-Lungen-Transplantation, weshalb sie laut den ISHLT-Konsensdokumenten (2014, 2021) die bevorzugte Methode für PAH-Patienten ist. In Fällen, in denen eine kardiale Ursache vorliegt, die operativ korrigiert werden kann, wird eine bilaterale Lungentransplantation mit chirurgischer Reparatur bevorzugt, anstelle einer kombinierten Herz-Lungen-Transplantation 17 . Eine Analyse von Patienten mit Eisenmenger-Syndrom zeigte, dass eine bilaterale Lungentransplantation mit Reparatur bei Vorliegen eines Vorhofseptumdefekts bessere Überlebensraten ergab. Hingegen war dies bei einem Ventrikelseptumdefekt nicht der Fall, was darauf hindeutet, dass in solchen komplexeren Fällen eine kombinierte Herz-Lungen-Transplantation in Erwägung gezogen werden kann 18 .

Obwohl die Einführung des Lung Allocation Score (LAS) weitgehend als Erfolg gewertet wird, hatte sie unbeabsichtigte Folgen für Patienten mit pulmonaler arterieller Hypertonie (PAH). Die zur Berechnung des LAS herangezogenen Parameter – wie forcierte Vitalkapazitat (FVC), Sauerstoffbedarf und Anstieg des Kohlendioxidgehalts – sind bei PAH häufig nur geringfügig verändert, was dazu führt, dass diese Patienten im Verhältnis zur Schwere ihrer Erkrankung einen vergleichsweise niedrigen LAS erhalten. Pulmonale arterielle Hypertonie war die einzige Krankheitsgruppe, bei der die Mortalität auf der Warteliste nach Einführung des LAS nicht gesenkt wurde 19 . Tatsächlich waren PAH-Patienten nach der Einführung des LAS die Gruppe, die am wahrscheinlichsten auf der Warteliste starb und am wenigsten wahrscheinlich transplantiert wurde.

Trotz der Seltenheit als Diagnosegrund für eine Transplantation stellen PAH-Patienten die Mehrheit der Ausnahmeanträge (Exceptional Lung Allocation Score, eLAS). Bemerkenswert ist, dass Patienten mit PAH, deren Ausnahmeanträge abgelehnt wurden, eher auf der Warteliste sterben als diejenigen, deren Antrag genehmigt wurde – ein Unterschied, der bei anderen Diagnosen nicht zu beobachten ist.

In der Zukunft könnte die Kombination aus einem verfeinerten LAS (Lung Composite Allocation Score), der ebenfalls eine Vorimmunisierung berücksichtigt, und neuen gezielten Therapien wie Sotatercept nicht nur die Präzision bei der Zuteilung von Transplantationen erhöhen, sondern auch die Notwendigkeit einer Transplantation bei gut kontrollierter PAH reduzieren 20 21 .

## Retransplantationen

Basierend auf der ISHLT-Registry wurden 1597 Patienten retransplantiert. Seit 2005 machten Retransplantationen etwa 4–6% der Transplantationen aus. Rund 68% der Retransplantationen erfolgten mehr als 2 Jahre nach der ersten Transplantation, 9,4% innerhalb der ersten 90 Tage. Das 1-Jahres-Überleben lag bei 73,8%. Eine große retrospektive Analyse identifizierte 3 wichtige Risikofaktoren für die Mortalität im 1. Jahr: das Intervall zwischen den Transplantationen, das Alter und die Notwendigkeit einer mechanischen Ventilation vor der Retransplantation. Patienten, die bei beiden Transplantationen eine bilaterale Transplantation erhielten, hatten eine bessere Überlebensrate als diejenigen, die eine Einzeltransplantation erhielten 22 .

Die Studie betont die Bedeutung von Interzentrumsunterschieden und schlägt vor, spezifische Allokationsscores für Retransplantationen zu entwickeln. Insgesamt zeigen die Ergebnisse, dass Lungentransplantationen bei Retransplantationen eine zufriedenstellende, jedoch geringere Überlebensrate im Vergleich zu Ersttransplantationen aufweisen. Zukünftige Studien und kontinuierliche Datensammlung sind entscheidend, um die besten Praktiken für Retransplantationen zu identifizieren 22 .

## Fazit

Abschließend lässt sich sagen, dass die 2021 aktualisierten ISHLT-Richtlinien den Fortschritt in der Lungentransplantation der letzten Jahrzehnte widerspiegeln. Seit den aktualisierten Richtlinien wurden in der klinischen Praxis zunehmend Risikofaktoren berücksichtigt, die Indikationen erweitert und ein individualisierter Ansatz verfolgt, was dazu beigetragen hat, Lungentransplantationen für immer mehr Patienten zu einer realistischen Option zu machen. Neben dem medizinischen Erfolg sollte jedoch auch die Lebensqualität der Patienten nach der Transplantation sowie die psychosozialen Aspekte nicht unbeachtet bleiben. Viele Patienten erleben eine Verbesserung der Lebensqualität, aber Herausforderungen wie Ängste vor Abstoßung, Infektionen und die Anpassung an die Langzeitmedikation sind ebenfalls häufig. Psychosoziale Unterstützung durch ein interdisziplinäres Team ist entscheidend, um das Wohlbefinden der Patienten langfristig zu fördern.
